# CRTC2 enhances HBV transcription and replication by inducing PGC1α expression

**DOI:** 10.1186/1743-422X-11-30

**Published:** 2014-02-14

**Authors:** Xiaohui Tian, Fei Zhao, Weihua Sun, Xiaoguang Zhi, Zhikui Cheng, Ming Zhou, Kanghong Hu

**Affiliations:** 1 State Key Laboratory of Virology, Wuhan Institute of Virology, Chinese Academy of Sciences, Xiaohongshan Zhongqu 44, Wuhan 430071, China; 2 Biomedical Center, Hubei University of Technology, Wuhan 430068, China

**Keywords:** HBV, CRTC2, Forskolin, Phosphorylation-defective CRTC2 mutant, PGC1α

## Abstract

**Background:**

Hepatitis B virus (HBV) transcription and replication are essentially restricted to hepatocytes. Based on the HBV enhancer and promoter complex that links hepatic glucose metabolism to its transcription and replication, HBV adopts a regulatory system that is unique to the hepatic gluconeogenic genes. CRTC2, the CREB-regulated transcription coactivator 2, is a critical switch modulating the gluconeogenic program in response to both hormonal and intracellular signals. However, the relationship between CRTC2 and HBV transcription and replication remains unclear.

**Methods:**

To analyze the influence of CRTC2 on HBV transcription and replication, CRTC2 expression construct or siRNA was cotransfected with plasmids containing enhancer II/core promoter complex-controlled luciferase or 1.3× wtHBV genome in Huh-7 cells. Luciferase activity, HBV core protein expression, HBV transcripts, and DNA replication intermediates were measured by luciferase assays, western blots, real-time polymerase chain reaction (PCR), and Southern blots, respectively. Forskolin (FSK) or phosphorylation-defective CRTC2 mutants were further utilized to elucidate the potential mechanism. siRNA against peroxisome proliferator-activated receptor-γ coactivator 1α (PGC1α) was also used to examine the mediator involved in CRTC2-regulated HBV biosynthesis in Huh-7 cells.

**Results:**

CRTC2 overexpression increased HBV transcription and replication in Huh-7 cells, including levels of core protein expression, mRNA, and DNA replication intermediates. Correspondingly, CRTC2 knock down by siRNA reduced HBV biosynthesis. FSK treatment strongly enhanced the effect of CRTC2 through triggering the dephosphorylation and nuclear entry of CRTC2. The phosphorylation-defective mutant (S171A/S275A) of CRTC2 localized in the nucleus and was constitutively active, which dramatically promoted HBV transcription and replication similar to FSK-treated wild-type CRTC2. Knock down of PGC1α, whose expression was induced by CRTC2, greatly compromised the enhancing effect of CRTC2 on HBV transcription and replication.

**Conclusions:**

Our results clearly indicate that non-phosphorylated CRTC2 strongly enhances HBV biosynthesis through inducing PGC1α expression. Further study of the mechanisms will elucidate the importance of metabolic signals on HBV transcription and replication, and offer insight into potential targets for developing anti-HBV agents.

## Background

Hepatitis B virus (HBV) is the prototypical member of hepadnaviruses, a small enveloped DNA virus family preferentially targeting the liver [1-3]. Multiple studies have implicated that HBV transcription and replication might be regulated by transcription factors capable of mediating hepatic gluconeogenesis [2,4]. The cAMP-responsive element-binding protein (CREB)-regulated transcription coactivator 2 (CRTC2) is a critical switch promoting the gluconeogenic program in the liver [5]. Under normal physiological conditions, CRTC2 is phosphorylated at Ser171 and Ser275 by SIK1 (salt-inducible kinase 1) and MARK2 (microtubule affinity-regulating kinase 2), respectively. Phosphorylation of CRTC2 triggers phosphorylation-dependent binding to 14-3-3 proteins, which sequesters CRTC2 in the cytoplasm [6,7]. In response to fasting, CRTC2 is dephosphorylated and translocates into the nucleus, where it coactivates CREB and further stimulates the expression of downstream gluconeogenic genes [5]. The dephosphorylation of CRTC2 at Ser171 and Ser275 is triggered by the calcium-regulated Ser/Thr phosphatase calcineurin following exposure to cAMP and calcium [5,10]. Thus, the dephosphorylated and nuclear-translocated CRTC2 is considered to be the active form [8]. Forskolin (FSK) treatment dramatically raises cellular cAMP and calcium levels and dephosphorylates CRTC2, which renders it active by allowing it to translocate to the nucleus [10]. The calcineurin inhibitor cyclosporine A (CsA) disrupts the cAMP-induced dephosphorylation and nuclear translocation of CRTC2, but it has no effect on a phosphorylation-defective CRTC2 mutant (S171A/S275A) [8]. Because it cannot be phosphorylated at these two sites, the S171A/S275A mutant remains active in the nucleus [11,12].

The HBV genome comprises a partially double-stranded DNA that binds a variety of liver-enriched nuclear receptors, some of which are also critical regulators implicated in gluconeogenesis [4,6,7,13,14]. As a coactivator of key gluconeogenesis genes, peroxisome proliferator-activated receptor-γ coactivator 1α (PGC1α) expression is also stimulated by active CRTC2. It has been proved that PGC1α coactivates HBV transcription and replication through hepatocyte nuclear factor-4α (HNF4α) and forkhead transcription factor FOXO1, which has also been confirmed in our previously published data [15,16]. Thus, we sought to study the link between HBV transcription activity and hepatic metabolic state [16-20], especially the unknown relationship of CRTC2 with HBV biosynthesis.

To address these issues, HBV transcription and replication activities were analyzed by measuring enhancer II/core promoter complex-controlled luciferase activity, core protein expression, mRNA levels, and DNA replication intermediates in Huh-7 cells transfected with HBV constructs together with the indicated constructs. FSK and phosphorylation-defective CRTC2 mutants were also employed to elucidate the potential mechanism of CRTC2’s effects on HBV transcription and replication. Our results showed that CRTC2 increased HBV biosynthesis, and this effect was dramatically promoted by Ser171 and Ser275 dephosphorylation, which stimulated CRTC2 nuclear translocation and transformed CRTC2 into the active form. Importantly, the enhancement of CRTC2 on HBV transcription and replication was mediated by inducing PGC1α expression. Considering the critical roles of CRTC2 and PGC1α in the gluconeogenic program, our findings indicate the importance of understanding the regulatory effects of metabolic signals on HBV transcription and replication, which could offer insight into novel targets for anti-HBV therapy.

## Results

### CRTC2 enhances HBV transcription

**Figure 1:**
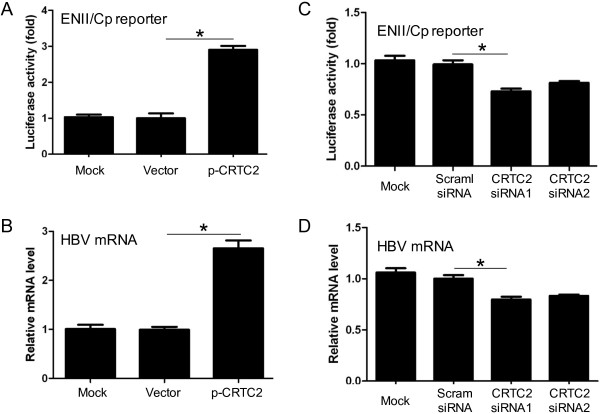
**CRTC2 enhances HBV transcription. (A)** Luciferase activity in Huh-7 cells transfected with pGL-3-ENII-luc and p-CRTC2. **(B)** HBV mRNA levels in Huh-7 cells transfected with 1.3× wtHBV plasmid and p-CRTC2. **(C-D)** The effect of CRTC2 knock down on luciferase activity **(C)** and HBV mRNA levels **(D)** in pGL-3-ENII-luc and 1.3× wtHBV plasmid-transfected Huh-7 cells, respectively. *p < 0.05.

Due to its enhancer and promoter composition, HBV transcription and replication can be regulated by nutritional signals that control hepatic glucose metabolism [2]. As a critical switch promoting the hepatic gluconeogenic program, CRTC2 attracted our interest for its role in HBV transcription and replication. The enhancer II/core promoter sequence regulates the transcription of 3.5-kb pregenomic RNA (pgRNA) that initiates the key step for HBV replication [6,21], so we first examined the effect of CRTC2 overexpression on the enhancer II/core promoter. For this aim, CRTC2 plasmid (p-CRTC2) was transfected into Huh-7 cells together with pGL-3-ENII-luc, a plasmid that expressed luciferase under the control of HBV enhancer II/core promoter complex. CRTC2 expression in transfected Huh-7 cells was confirmed by western blot analysis detecting the FLAG-tag (Additional file 1: Figure S1A). The results presented in Figure 1A clearly showed elevated luciferase activity in the presence of overexpressed CRTC2 in Huh-7 cells, indicating that transfected CRTC2 activated the enhancer II/core promoter complex. In 1.3× wtHBV plasmid-transfected Huh-7 cells, exogenous CRTC2 expression also increased HBV transcripts (Figure 1B). Two different siRNAs against CRTC2 and control siRNA were applied to investigate the effect of endogenous CRTC2 on HBV transcription. Both siRNAs against CRTC2 clearly reduced CRTC2 protein levels, but not the control siRNA (Additional file 1: Figure S1B). CRTC2 knock down by siRNA significantly reduced enhancer II/core promoter-controlled luciferase expression (Figure 1C) and HBV transcripts (Figure 1D), indicating the regulatory role of CRTC2 in HBV transcription. Because knock down of endogenous CRTC2 only led to a mild reduction in HBV transcription, FSK and exogenous CRTC2 were further applied to explore the potential mechanism involved in CRTC2 enhanced HBV transcription and replication.

### FSK robustly enhances CRTC2-induced HBV transcription and replication

**Figure 2:**
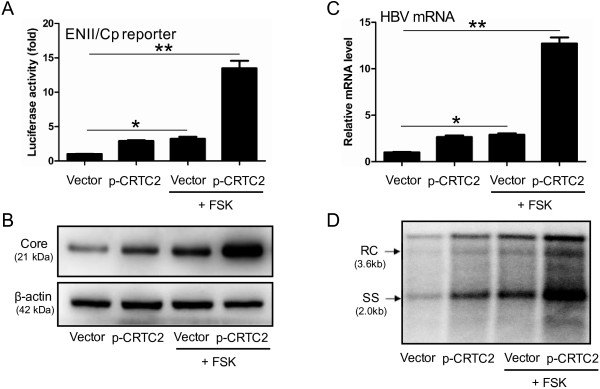
**FSK robustly enhances CRTC2-induced HBV transcription and replication. (A)** Luciferase activity in Huh-7 cells transfected with pGL-3-ENII-luc and p-CRTC2 in the presence or absence of FSK. **(B-D)** HBV core protein expression **(B)**, HBV mRNA levels **(C)**, and HBV DNA replication intermediates **(D)** in Huh-7 cells transfected with p-CRTC2 and 1.3× wtHBV plasmid, in the presence or absence of FSK. *p < 0.05; **p < 0.01.

As a cAMP inducer, FSK strongly promotes CRTC2 dephosphorylation and nuclear translocation [5], which stimulates downstream gene expression [17,22]. CRTC2 was primarily localized in the cytoplasm in the absence of FSK, and upon FSK treatment, it was dephosphorylated (Additional file 2: Figure S2B) and translocated into the nucleus (Additional file 2: Figure S2A). Considering the active form of nuclear-translocated CRTC2 [5], we examined whether FSK treatment influenced its regulatory role in HBV transcription and replication. Huh-7 cells were treated with 10 μM FSK 16 hours post-transfection with pGL-3-ENII-luc and/or p-CRTC2. In accordance with our previously described results, CRTC2 had marginal effects on luciferase activity. Notably, this effect was robustly promoted by FSK treatment (Figure 2A). Similar FSK-induced enhancements were observed for core protein expression (Figure 2B), HBV transcript levels (Figure 2C), and DNA replication intermediates (Figure 2D) in Huh-7 cell transfected with 1.3× wtHBV construct. Even in the absence of exogenous CRTC2, FSK treatment increased HBV biosynthesis to some extent (Figure 2A-D), possibly due to the existence of endogenous CRTC2. However, the basal level of endogenous CRTC2 only exerted a mild increasing effect, which was completely abrogated by treatment with CRTC2 siRNA (Additional file 3: Figure S3). Overall, these results indicate that FSK dramatically enhanced CRTC2-induced HBV transcription and replication.

### Phosphorylation-defective CRTC2 mutants strongly upregulate HBV biosynthesis

**Figure 3:**
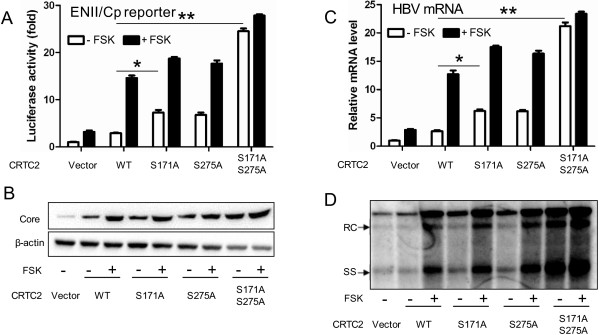
**Phosphorylation-defective CRTC2 mutants strongly upregulate HBV biosynthesis. (A)** The effect of a phosphorylation-defective mutation of CRTC2 (S171A, S275A, and S171A/S275A) on luciferase activity in pGL-3-ENII-luc transfected Huh-7 cells with or without FSK. **(B-D)** HBV core protein expression **(B)**, HBV mRNA level **(C)**, and HBV DNA replication intermediates **(D)** in Huh-7 cells transfected with 1.3× wtHBV plasmid and wild-type or mutated CRTC2 in the presence or absence of FSK. *p < 0.05; **p < 0.01.

Under basal conditions, CRTC2 is sequestered in the cytoplasm through phosphorylation at Ser171 and Ser275 [5,23]. Nuclear translocation and subsequent downstream gene transcription requires dephosphorylating at these two sites [8,24]. Mutations at individual (Ser171Ala, Ser275Ala) or both sites (Ser171Ala/Ser275Ala) were carried out to generate phosphorylation-defective mutants (Additional file 2: Figure S2B), which could not be phosphorylated and primarily localized in the nucleus (Additional file 2: Figure S2A). To investigate whether FSK enhanced CRTC2-induced HBV biosynthesis through dephosphorylating Ser171 and Ser275, phosphorylation-defective mutant constructs were transfected into Huh-7 cells, together with pGL-3-ENII-luc (Figure 3A) or 1.3× wtHBV construct (Figure 3B-D). Compared to wild-type CRTC2, single-site mutation (S171A or S275A) significantly increased luciferase activity (Figure 3A), core protein expression (Figure 3B), HBV transcript levels (Figure 3C), and the number of DNA replication intermediates (Figure 3D). Notably, phosphorylation-defective mutations at both sites (S171A/S275A) induced HBV biosynthesis to the greatest degree (Figure 3A-D). FSK treatment significantly promoted the increasing effect of wild-type CRTC2 and both single-site mutants of CRTC2 (S171A or S275A), but it only slightly enhanced activity of the double-site mutated CRTC2 (S171A/S275A) (Figure 3A-D), which was possibly due to activity of the endogenous wild-type CRTC2. These results indicate that FSK enhanced CRTC2-induced HBV biosynthesis by dephosphorylating Ser171/Ser275 and triggering CRTC2 nuclear entry.

### CRTC2 enhances HBV biosynthesis through inducing PGC1α expression

**Figure 4:**
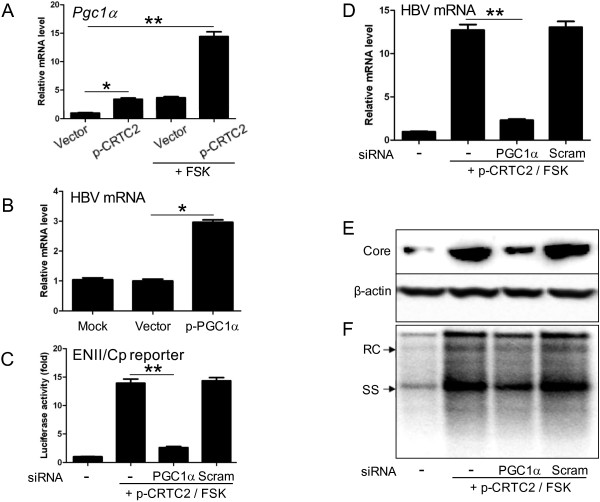
**CRTC2 enhances HBV replication by inducing PGC1α expression. (A)** PGC1α expression level in Huh-7 cells transfected with p-CRTC2 with or without FSK treatment. **(B)** HBV transcript amounts in Huh-7 cells transfected with 1.3× wtHBV plasmid and p-PGC1α. **(C)** Effect of PGC1α knock down on luciferase activity in Huh-7 cells transfected with pGL-3-ENII-luc and p-CRTC2 with FSK treatment. **(D-F)** Effect of PGC1α knock down on HBV mRNA level **(D)**, HBV core protein expression **(E)**, and HBV DNA replication intermediates **(F)** in Huh-7 cells transfected with 1.3× wtHBV plasmid and p-CRTC2 with FSK treatment. *p < 0.05; **p < 0.01.

PGC1α is one of the downstream genes stimulated by CRTC2 [25,26]. Our data clearly showed that CRTC2 induced PGC1α mRNA expression, which was further significantly promoted by FSK (Figure 4A). In accordance with previously published data [15,17], exogenous PGC1α expression significantly upregulated HBV transcription (Figure 4B). Therefore, we investigated whether CRTC2-enhanced HBV biosynthesis was mediated by PGC1α expression. To address this issue, siRNA was applied to knock-down PGC1α expression in Huh-7 cells transfected with p-CRTC2 and pGL-3-ENII-luc (Figure 4C) or 1.3× wtHBV (Figure 4D-F). The knock down efficiency of PGC1α was confirmed by quantitative PCR (Additional file 4: Figure S4A and B). As previously shown, CRTC2 strongly upregulated HBV biosynthesis following FSK treatment. However, PGC1α knock down significantly attenuated the enhancing effect of CRTC2/FSK on luciferase activity (Figure 4C), HBV transcript levels (Figure 4D), core protein expression (Figure 4E), and DNA replication intermediates (Figure 4F). These results suggest that CRTC2 enhancing HBV biosynthesis was dependent on the induction of PGC1α expression.

## Discussion

The CRTC family consists of three isoforms (CRTC1, CRTC2 and CRTC3), all of which can act as CREB coactivators and potentiate CREB-dependent transcription. CRTC1 and CRTC3 play important roles in energy balance [22], whereas CRTC2 induces the gluconeogenic program and thus promotes glucose homeostasis during fasting in liver [5]. It has been shown that HBV biosynthesis can be regulated by signals involved in the gluconeogenic program. Thus, we were interested in investigating whether the hormonal and energy-sensing coactivator CRTC2 influenced HBV transcription and replication.

In response to FSK, CRTC2 is dephosphorylated and transported to the nucleus, which is a critical step in stimulating downstream gene expression, including that of PGC1α [5,27]. CRTC2 promotes PGC1α expression through its role as a coactivator of CREB [8]. The CREB-CRTC pathway triggered by hormonal and nutrient signals has been extensively characterized, and the CREB-CRTC/PGC1α signal transduction pathway has been shown to mediate glucose homeostasis in the liver [8,28,29]. In addition, the effect of the insulin signaling pathway on HBV pgRNA synthesis and viral replication has been proven to be PGC1α dependent [20]. Collectively, these observations have led to the suggestion that the regulation of HBV transcription and replication may utilize similar signal transduction pathways as those involved in the expression of key hepatic metabolic genes [17,30-32].

Our results clearly demonstrated that HBV transcription and replication was regulated by CRTC2. Overexpression of exogenous CRTC2 increased HBV biosynthesis in Huh-7 cells, whereas its knock down had the opposite effect. Hepatic CRTC2 is regulated through phosphorylation at Ser171 and Ser275 by SIKs and members of the AMP-activated protein kinase (AMPK), such as MARK2 [22]. As a result, phosphorylated CRTC2 was sequestered in the cytoplasm through phosphorylation-dependent interactions with 14-3-3 proteins [5,22]. FSK treatment significantly enhanced CRTC2-induced HBV transcription and replication, which was mediated by dephosphorylating CRTC2 and triggering its nuclear translocation. Interestingly, phosphorylation-defective mutants with single-site mutations (S171A or S275A) showed only mild enhancement compared to wild-type CRTC2, which was possibly due to the remaining phosphorylation site. FSK application further promoted the increasing effect of S171A or S275A. In accordance with the above-mentioned hypothesis, CRTC2 mutant S171A/S275A with both two defective phosphorylation sites dramatically increased HBV transcription and replication, but FSK did not have a significant effect on S171A/S275A-induced activity. The slight increase of S171A/S275A-induced HBV biosynthesis following FSK treatment might be explained by the presence of endogenous wild-type CRTC2, which could still be affected by FSK. Most current anti-HBV drugs are nucleotide/nucleoside analogs, which are double-edged swords given the emergence of drug-resistant HBV mutants and potential disease flare-ups [19]. Indeed, nucleotide/nucleoside analogs fail to eliminate the HBV cccDNA pool and usually require prolonged or life-long treatment [19]. Therefore, the ideal therapy should combine both virus-specific and host-directed treatments. Our results clearly showed that the glucose homeostasis regulator CRTC2 has the potential to modulate HBV transcription and replication in Huh-7 cells, suggesting that viral biosynthesis may be regulated by hepatic metabolic state. The observation that diabetic patients are more likely to suffer acute HBV infections than non-diabetic patients supports the contention that metabolic signaling through CRTC2 can influence the outcome of viral infection [33,34]. To mimic the physiological starving condition, glucose-free media was applied in our preliminary experiments, but transfected cells failed to survive under these conditions. For this reason, FSK, which was previously shown to simulate a starvation state, was applied to cells as an alternative approach [18]. Under feeding conditions, CRTC2 is sequestered in the cytoplasm, and fasting or FSK treatment dephosphorylated CRTC2 and transported it to the nucleus [22], where it enhanced HBV transcription and replication. Our work builds on the elaborate and exciting report published by Shaul’s group, which clearly demonstrated that PGC1α regulates HBV transcription and replication in response to nutritional cues [17]. Collectively, our data and published reports concerning HBV and nutrition conditions suggest that HBV patients should completely avoid food deprivation conditions. It has also been reported that malnutrition might be a key element responsible for HBV infection [2]. Thus, proper nutrition, including the digestion of complex carbohydrates to avoid hypoglycemia that results in increased glucagon levels, may be a critical component of novel anti-HBV therapeutic strategies. Concerning the dependency of HBV transcription and replication on momentary nutritional status, the optimal time to collect blood samples from patients for HBV copy number testing should be also taken into consideration.

Elucidating the regulatory role of the CRTC2/PGC1α signaling pathway and the underlying mechanism will reveal important molecular processes involved in HBV biosynthesis and elucidate the relationship between metabolic signals and HBV replication during natural infection. Such knowledge could contribute to the development of novel therapeutic approaches.

## Conclusions

In summary, our results demonstrate a novel up-regulatory role of CRTC2 on HBV biosynthesis. CRTC2 activated the enhancer II/Core promoter complex, increased core protein expression, and elevated HBV transcripts and DNA replication intermediates. This enhancing effect was dramatically promoted by CRTC2 dephosphorylation. Importantly, CRTC2 enhanced HBV transcription and replication by inducing PGC1α expression.

## Materials and methods

### Plasmid constructs

The 1.3× wtHBV plasmid (1.3× wtHBV) encoding 1.3 copies of the HBV genome (*adw* strain) was kindly provided by Dr. Yosef Shaul [6]. The pGL-3-ENII-luc construct expressing luciferase under the control of HBV ENII/core promoter complex (nt.1403–1803) was a gift from Dr. Jing-hsiung James Ou [35]. The p-CRTC2 plasmid was constructed by inserting wild-type human CRTC2 cDNA into pCDNA3.1 FLAG + and the phosphorylation-defective CRTC2 mutants (S171A, S275A, and S171A/S275A) were generated by site-directed mutagenesis based on the p-CRTC2 plasmid. pSuper vector [36] containing the human H1 promoter was used as the backbone to generate siRNA expression plasmids against CRTC2 and PGC1α. The applied siRNA sequences were CRTC2 siRNA1 5′- gcc caa tgt taa cca gat t -3′, siRNA2 5′- cag cga gat cct cga aga atg-3′ and PGC1α siRNA 5′- ggt gga ttg aag tgg tgt a -3′ [17].

### Cell culture and transfection

The human hepatoma cell line Huh-7 were cultured in Dulbecco’s modified Eagle’s medium supplemented with 10% (vol/vol) fetal bovine serum, 100 U/ml penicillin and 100 μg/ml streptomycin (all from Gibco Life Technologies, USA) at 37ºC under a 5% CO_2_ atmosphere. Transfection was performed in 10 cm cell culture dishes containing 1 × 10^6^ Huh-7 cells by using Lipofectamine 2000 according to the manufacturer’s instructions (Invitrogen, USA). The following constructs were applied for transfection: 1.3× wtHBV (3 μg), pGL-3-ENII-luc (3 μg), p-CRTC2 (9 μg), p-CRTC2 (S171A) (9 μg), p-CRTC2 (S275A) (9 μg), p-CRTC2 (S171A/S275A) (9 μg), CRTC2 siRNA (9 μg), and PGC1α siRNA (9 μg). The same amount of vector lacking the corresponding insert was used as controls. When indicated, cells were treated with 10 μM FSK (Sigma, USA) 16 hours post-transfection.

### Quantitative real-time PCR

HBV transcript and PGC1α expression analyses were performed as described previously [17]. Briefly, transfected cells were homogenized in TRIzol (Invitrogen, USA) for total RNA isolation, and cDNA were synthesized using PremeScript RT reagent kit (Takara, Japan) following the manufacturer’s instructions. Quantitative real-time PCR was performed using SYBR Green Realtime PCR Master Mix (Toyobo, Japan) in the Mx3000P qPCR System (Agilent Technologies, USA). The results were normalized to the housekeeping gene β-actin in all assays. The following primers were used: HBV, 5′- ctc agc tct gta tcg aga agc c -3′ (sense) and 5′- cag tga gag ggc cca caa att g -3′ (antisense); PGC1α, 5′- tcc tca cag aga cac tag aca g -3′ (sense) and 5′- ctg gtg cca gta aga gct tct -3′ (antisense); β-actin, 5′- acc gcg aga aga tga ccc ag -3′ (sense) and 5′- cca tct cgt tct cga agt cca -3′ (antisense).

### Southern blot analysis

Encapsidated HBV DNA was isolated using the method described by Guo [37]. Briefly, 1 × 10^6^ transfected cells were collected 3 days post-transfection and lysed in 300 μl lysis buffer (50 mM Tris–HCl [pH 7.4], 1 mM EDTA, and 1% NP-40). After centrifugation, the supernatant was treated with DNase I (100 μg/ml) and RNase A (100 μg/ml) for 30 minutes at 37°C to remove the extra-capsid nuclear acid. HBV DNA in the intracellular core particles was isolated with phenol-chloroform (1:1) extraction and ethanol precipitation after being released by digesting capsid proteins with proteinase K in the presence of 1% sodium dodecyl sulfate (SDS) for 2 hours. Following electrophoresis in a 1.0% agarose gel, encapsidated HBV DNA was blotted onto a nylon membrane and hybridized with a ^32^P-labeled HBV-specific probe. Signals were visualized using Cyclone Plus Storage Phosphor System (Perkin Elmer, USA).

### Western blot analysis

For protein analysis, 1 × 10^6^ transfected cells were lysed in 300 μl lysis buffer (50 mM Tris–HCl [pH 8.0], 150 mM NaCl, 0.1% SDS, 1% NP40, 0.5% deoxycholic acid, 0.5% sodium azide, and 100 μl/ml phenylmethanesulfonyl fluoride). The lysates of each sample were electrophoresed with 12% SDS-PAGE gel and blotted onto PVDF membranes (Immobilon P, Millipore, USA). The blotted membranes were then incubated with the indicated primary antibodies followed by the corresponding secondary horseradish peroxidase (HRP)-conjugated antibodies. Signals were visualized using a DAB Western Blot kit (Thermo Fisher Scientific, USA). The following antibodies were used, monoclonal rabbit anti-CRTC2 (Epitomics, USA), rabbit anti-phospho-CRTC2 (Ser171) (Bioss, Scotland), polyclonal rabbit anti-HBc (DAKO, Denmark), monoclonal mouse anti-FLAG (Invitrogen, USA), monoclonal mouse anti-β-actin (Interchim, France), and goat anti-mouse IgG and goat anti-rabbit IgG (Proteintech, USA).

### Luciferase assay

Huh-7 cells were transfected with pGL-3-ENII-luc together with the indicated constructs. Luciferase activity was measured 48 hours post-transfection using the Luciferase Assay System (Promega, USA) according to the manufacturer’s instructions. Results are shown as the activity fold increase of firefly luciferase against *Renilla* luciferase, which was used for transfection efficiency adjustments.

### Statistical analysis

All bar graphs are shown as the mean ± standard error (SE) from at least three independent experiments, and results shown for Southern or western blotting are representative images selected from more than three independent experiments. Statistical differences were assessed with unpaired two-tailed Student’s *t-*tests. Differences were considered to be statistically significant when *p* < 0.05.

## Abbreviations

HBV: Hepatitis B virus; CRTC2: Camp response element-binding protein (CREB)-regulated transcription coactivator 2; PGC1α: Peroxisome proliferator-activated receptor-γ coactivator 1α; CREB: Camp response element-binding protein; FSK: Forskolin.

## Competing interests

The authors declare that they have no competing interests. Additional files are available at Virology Journal’s website.

## Authors’ contributions

XT performed the experiments. XT and KH designed the research. FZ, WS, XZ, ZC, and MZ provided experimental support. XT and FZ drafted the manuscript. All authors read and approved the final manuscript for submission.

## Supplementary Material

Additional file 1: Figure S1 **(A)** CRTC2 expression in Huh-7 cells 48 hours post p-CRTC2 or mock transfection. CRTC2 expression was analyzed by detecting FLAG-tag. **(B)** Knock down efficiency of CRTC2 siRNA on endogenous CRTC2 protein level. Click here for file

Additional file 2: Figure S2 **(A)** Effect of FSK or phosphorylation-defective mutations (S171A/S275A) on CRTC2 localization in Huh-7 cells 24 hours post-transfection. CRTC2 was detected by FLAG-tag. **(B)** The phosphorylation level of CRTC2 in Huh-7 cells transfected with wildtype or phosphorylation-defective mutant of CRTC2 (S171A) in the presence or absence of FSK. Click here for file

Additional file 3: Figure S3 CRTC2 siRNA attenuated the FSK enhancing effect on luciferase activity controlled by Enhancer II/Core promoter in Huh-7 cells. *p < 0.05. Click here for file

Additional file 4: Figure S4 **(A)** Knock down efficiency of siRNA on endogenous PGC1α expression in Huh-7 cells. **(B)** Knock down efficiency of siRNA on CRTC2/FSK induced PGC1α expression in Huh-7 cells. *p < 0.01. Click here for file
