# Supplementary material for: CRTC2 enhances HBV transcription and replication by inducing PGC1α expression
Source: Virol J. 2014 Feb 14;11:30. doi: 10.1186/1743-422X-11-30 (PMC3940274; doi:10.1186/1743-422X-11-30)
Supplement: Additional file 1: Figure S1 — (A) CRTC2 expression in Huh-7 cells 48 hours post p-CRTC2 or mock transfection. CRTC2 expression was analyzed by detecting FLAG-tag. (B) Knock down efficiency of CRTC2 siRNA on endogenous CRTC2 protein level. [file 1743-422X-11-30-S1.pptx]

## Slide 1
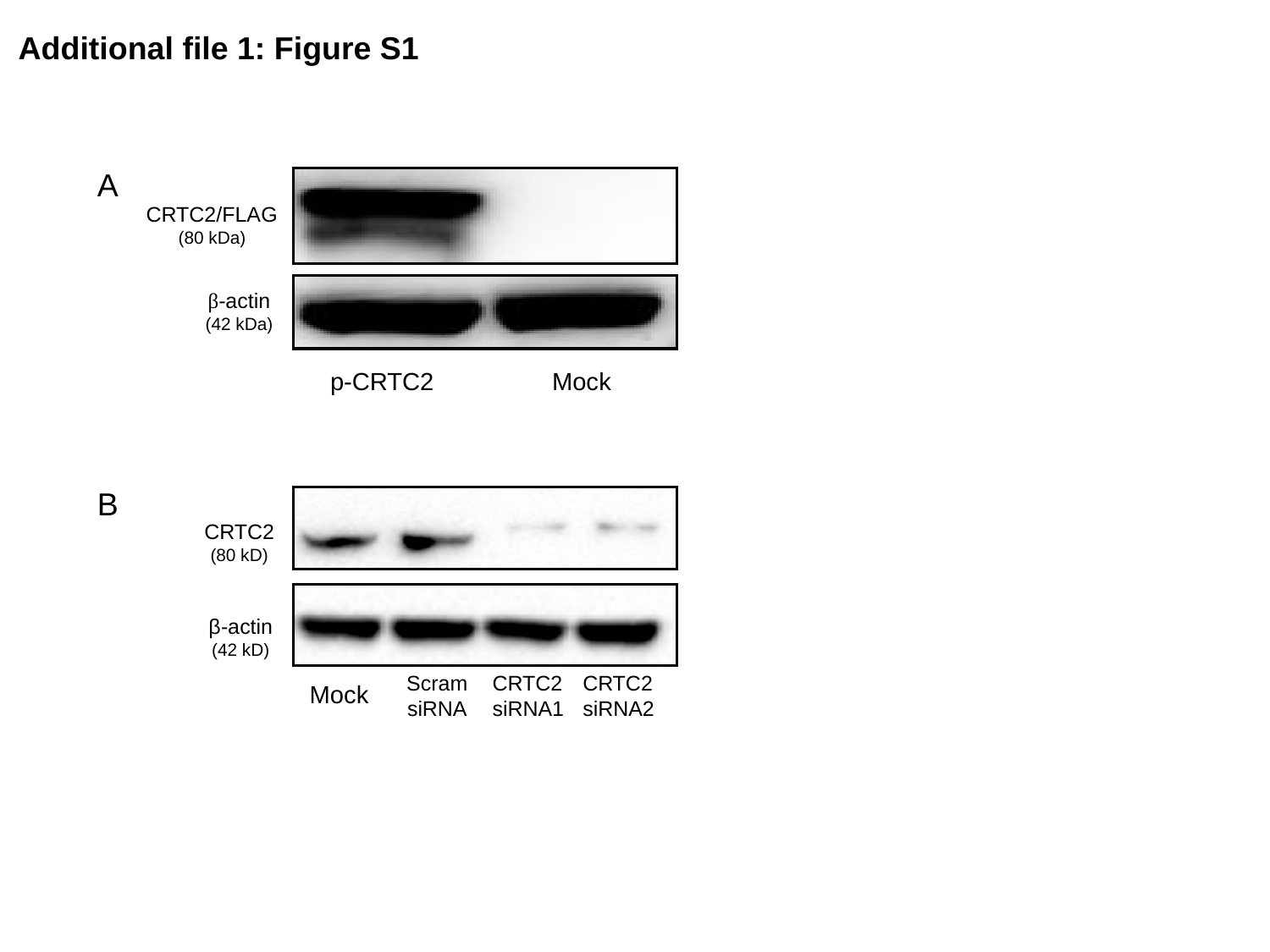

Additional file 1: Figure S1
A
CRTC2/FLAG
(80 kDa)
β-actin
 (42 kDa)
p-CRTC2
Mock
B
CRTC2
(80 kD)
β-actin
(42 kD)
Scram
siRNA
CRTC2
siRNA1
CRTC2
siRNA2
Mock
