# Supplementary material for: CRTC2 enhances HBV transcription and replication by inducing PGC1α expression
Source: Virol J. 2014 Feb 14;11:30. doi: 10.1186/1743-422X-11-30 (PMC3940274; doi:10.1186/1743-422X-11-30)
Supplement: Additional file 2: Figure S2 — (A) Effect of FSK or phosphorylation-defective mutations (S171A/S275A) on CRTC2 localization in Huh-7 cells 24 hours post-transfection. CRTC2 was detected by FLAG-tag. (B) The phosphorylation level of CRTC2 in Huh-7 cells transfected with wildtype or phosphorylation-defective mutant of CRTC2 (S171A) in the presence or absence of FSK. [file 1743-422X-11-30-S2.pptx]

## Slide 1
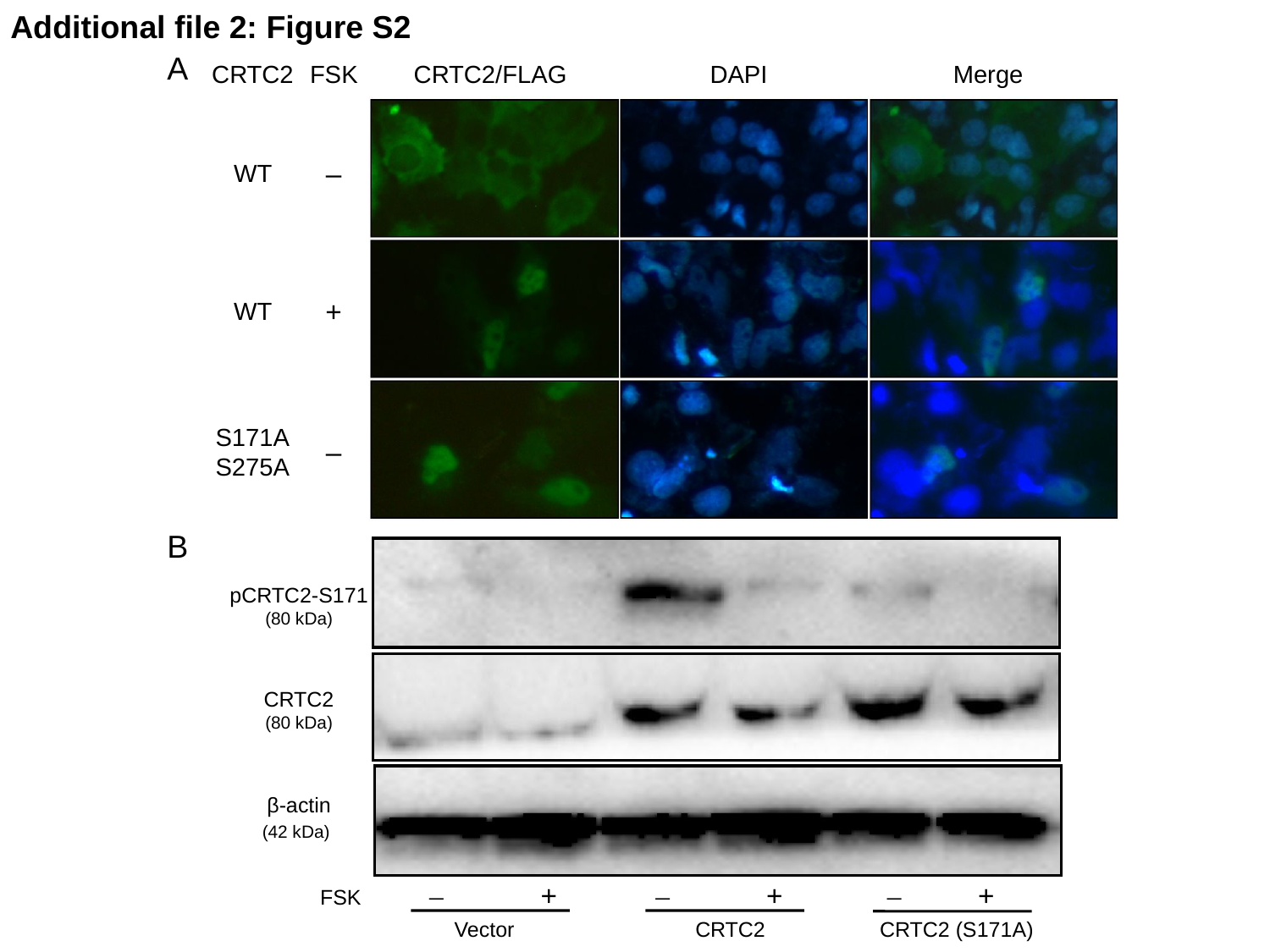

Additional file 2: Figure S2
A
CRTC2
FSK
CRTC2/FLAG
DAPI
Merge
–
WT
+
WT
S171A
S275A
–
B
pCRTC2-S171
(80 kDa)
CRTC2
(80 kDa)
β-actin
(42 kDa)
–
+
–
+
–
+
FSK
Vector
CRTC2
CRTC2 (S171A)
