# Supplementary figures and images for: CRTC2 enhances HBV transcription and replication by inducing PGC1α expression
Source: Virol J. 2014 Feb 14;11:30. doi: 10.1186/1743-422X-11-30 (PMC3940274; doi:10.1186/1743-422X-11-30)

## Slide 1
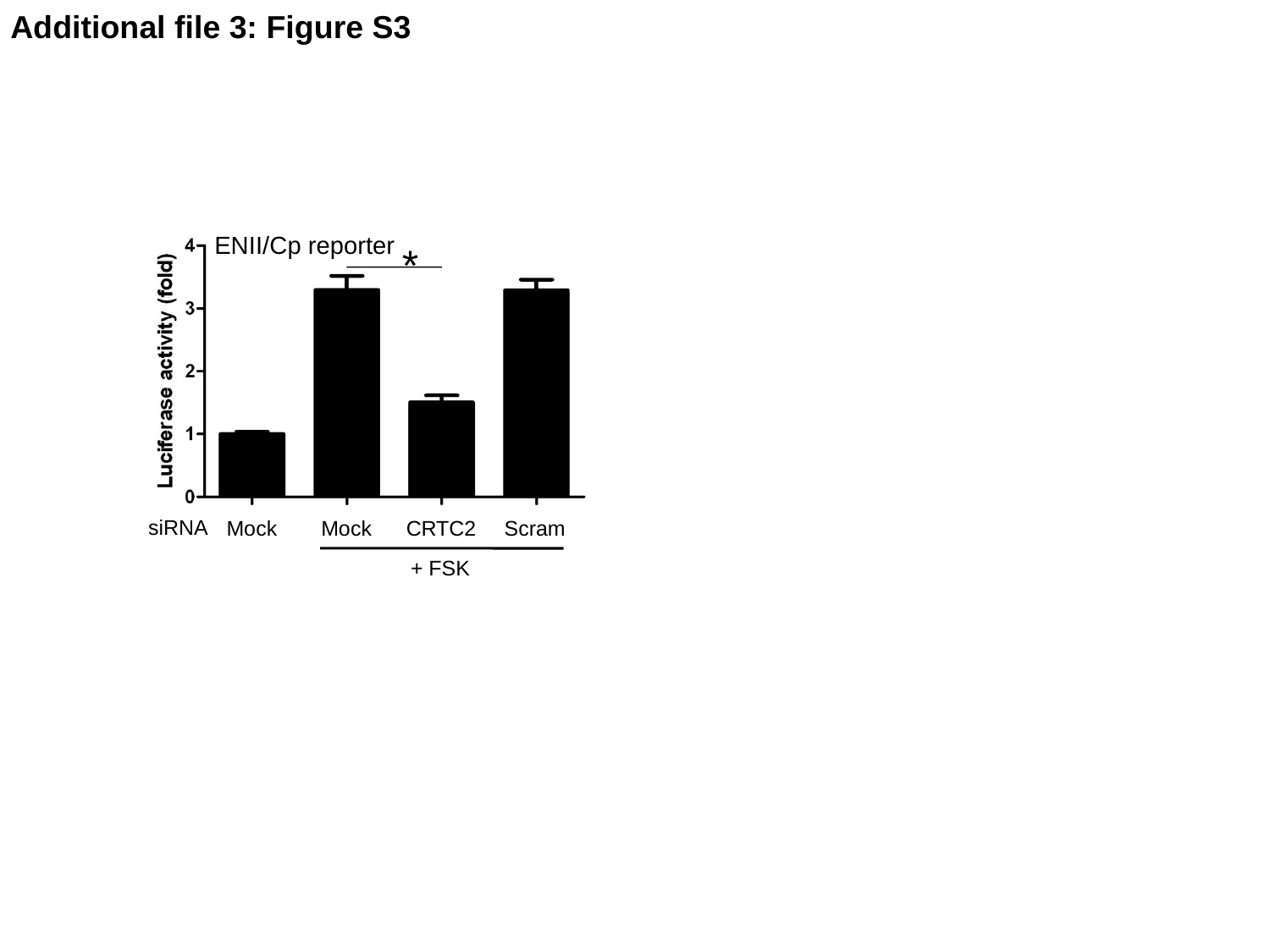

Additional file 3: Figure S3
ENII/Cp reporter
*
Mock
Mock
CRTC2
Scram
siRNA
+ FSK

Supplement: Additional file 3: Figure S3 — CRTC2 siRNA attenuated the FSK enhancing effect on luciferase activity controlled by Enhancer II/Core promoter in Huh-7 cells. *p < 0.05. [file 1743-422X-11-30-S3.pptx]
