# Supplementary material for: CRTC2 enhances HBV transcription and replication by inducing PGC1α expression
Source: Virol J. 2014 Feb 14;11:30. doi: 10.1186/1743-422X-11-30 (PMC3940274; doi:10.1186/1743-422X-11-30)
Supplement: Additional file 4: Figure S4 — (A) Knock down efficiency of siRNA on endogenous PGC1α expression in Huh-7 cells. (B) Knock down efficiency of siRNA on CRTC2/FSK induced PGC1α expression in Huh-7 cells. *p < 0.01. [file 1743-422X-11-30-S4.pptx]

## Slide 1
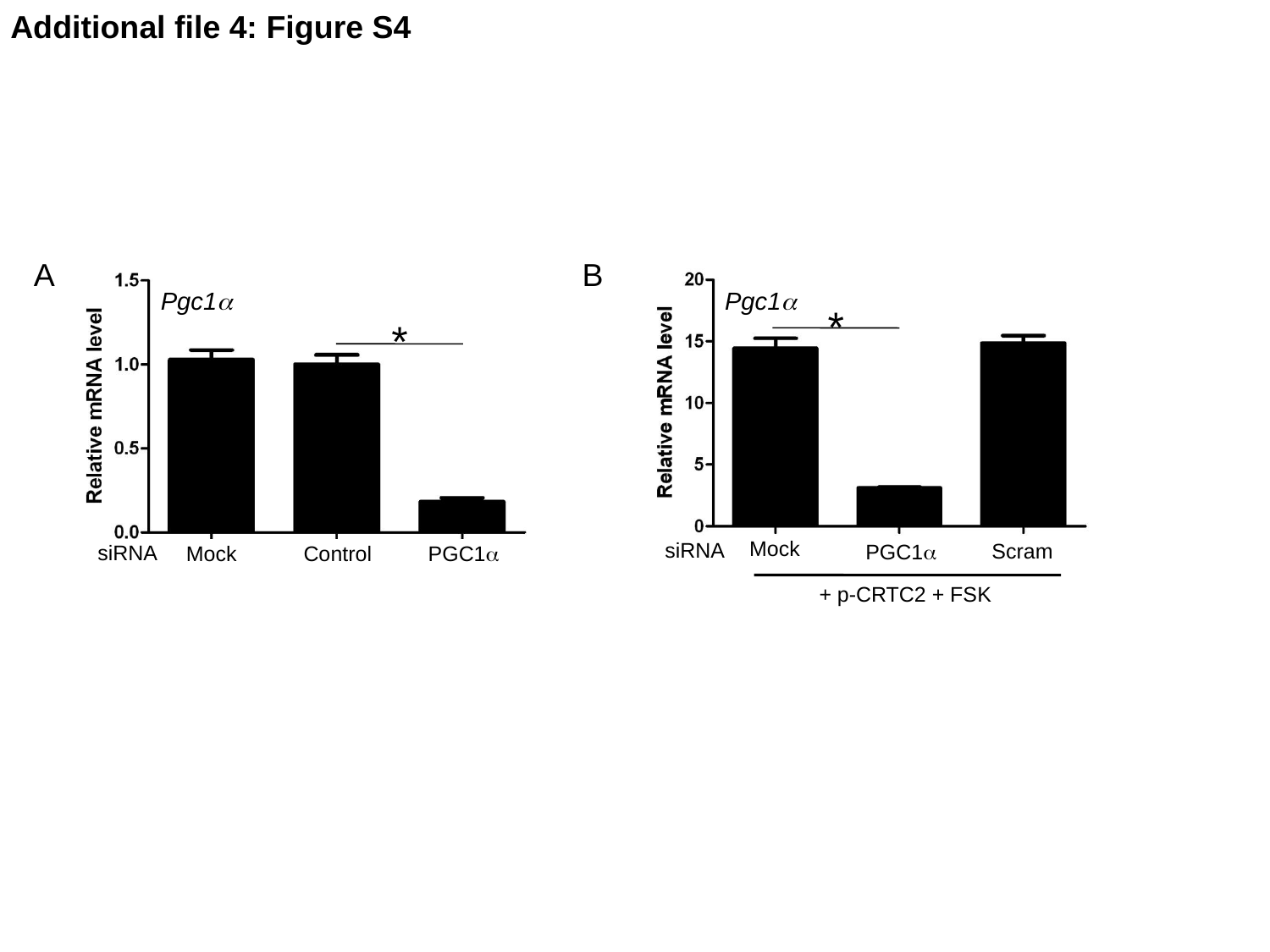

Additional file 4: Figure S4
A
B
Pgc1a
Pgc1a
*
*
Scram
siRNA
PGC1a
Mock
siRNA
Mock
Control
PGC1a
+ p-CRTC2 + FSK
